# The Role of Information Infrastructures in Scaling up Video Consultations During COVID-19: Mixed Methods Case Study Into Opportunity, Disruption, and Exposure

**DOI:** 10.2196/42431

**Published:** 2022-11-10

**Authors:** Joseph Wherton, Trisha Greenhalgh, Gemma Hughes, Sara E Shaw

**Affiliations:** 1 Nuffield Department of Primary Care Health Sciences University of Oxford Oxford United Kingdom

**Keywords:** information infrastructure, video consultations, organizational ethnography, COVID-19, health care organization, telehealth, health care delivery, agile governance, knowledge transfer

## Abstract

**Background:**

Until COVID-19, implementation and uptake of video consultations in health care was slow. However, the pandemic created a “burning platform” for scaling up such services. As health care organizations look to expand and maintain the use of video in the “new normal,” it is important to understand infrastructural influences and changes that emerged during the pandemic and that may influence sustainability going forward.

**Objective:**

This study aims to draw lessons from 4 National Health Service (NHS) organizations on how information infrastructures shaped, and were shaped by, the rapid scale-up of video consultations during COVID-19.

**Methods:**

A mixed methods case study of 4 NHS trusts in England was conducted before and during the pandemic. Data comprised 90 interviews with 49 participants (eg, clinicians, managers, administrators, and IT support), ethnographic field notes, and video consultation activity data. We sought examples of infrastructural features and challenges related to the rapid scale-up of video. Analysis was guided by Gkeredakis et al’s 3 perspectives on crisis and digital change: as opportunity (for accelerated innovation and removal of barriers to experimentation), disruption (to organizational practices, generating new dependencies and risks), and exposure (of vulnerabilities in both people and infrastructure).

**Results:**

Before COVID-19, there was a strong policy push for video consultations as a way of delivering health care efficiently. However, the spread of video was slow, and adopting clinicians described their use as ad hoc rather than business as usual. When the pandemic hit, video was rapidly scaled up. The most rapid increase in use was during the first month of the pandemic (March-April 2020), from an average of 8 video consultations per week to 171 per week at each site. Uptake continued to increase during the pandemic, averaging approximately 800 video consultations per week by March 2021. From an opportunity perspective, participants talked about changes to institutional elements of infrastructure, which had historically restricted the introduction and use of video. This was supported by an “organizing vision” for video, bringing legitimacy and support. Perspectives on disruption centered on changes to social, technical, and material work environments and the emergence of new patterns of action. Retaining positive elements of such change required a judicious balance between managerial (top-down) and emergent (bottom-up) approaches. Perspectives on exposure foregrounded social and technical impediments to video consulting. This highlighted the need to attend to the materiality and dependability of the installed base, as well as the social and cultural context of use.

**Conclusions:**

For sustained adoption at scale, health care organizations need to enable incremental systemic change and flexibility through agile governance and knowledge transfer pathways, support process multiplicity within virtual clinic workflows, attend to the materiality and dependability of the IT infrastructure within and beyond organizational boundaries, and maintain an overall narrative within which the continued use of video can be framed.

## Introduction

### Video Consulting Before, During, and Beyond the Pandemic

There has been growing interest in the use of video as a method of consultation between clinician and patient over the past 10 years, and numerous studies have shown such consultations to be acceptable, safe, and effective in selected patients [1-6]. However, until the pandemic, the uptake of video in the National Health Service (NHS), as in health care organizations in many countries, was slow. Sustained adoption at scale requires new ways of organizing clinical and administrative roles, new organizational routines, new approaches to privacy and information governance, and new clinical and communication skills. However, in published research trials of video consultations, the services were usually available only as part of the trial and discontinued thereafter, so the challenges of embedding video in business as usual were never addressed [7].

COVID-19 created a “burning platform” for the mainstreamed use of such services, as health care organizations worldwide halted face-to-face appointments for nonurgent care. The global emergency prompted strategic (policy decisions and legal changes), operational (increasing capacity and delivery by building skills and resources at pace and scale), and regulatory (eg, pandemic-related unofficial workarounds with unregulated products) changes with regard to the delivery of telehealth across different national contexts [8-10]. We initially flagged the pandemic as an “opportunity in a crisis” for giving video consulting the push it needed [11].

During the first wave of the pandemic in 2020, most countries saw a rapid reduction in face-to-face consultations and an increase in remote ones in both primary and secondary care [12-15]. Guidance for conducting video consultations were initially produced based on early examples of implementation [16-22]. More definitive guidance was later developed, based on learning during the pandemic, with the view of informing best practices beyond COVID-19 [23-26].

As health care organizations are looking to expand and maintain the use of video in the postpandemic “new normal,” it is important to acknowledge and harness wider system changes that have emerged. In this paper, we focus on the infrastructural aspects of the rapid scale-up of video and draw lessons for ongoing developments and sustainability. To this end, we explore the impact of the pandemic in 4 NHS organizations, where we had previously identified ways in which infrastructural features influenced the limited use of video in the years preceding the pandemic [27].

### Attending to the Health IT Infrastructure

Star [28] defines infrastructure as the stuff other things run on. It consists of hardware and software, the buildings, wires, connections, clinical records, organizational routines, standards, and other aspects that make an information system work. A defining characteristic of infrastructure is its transparency (invisible, taken for granted, and ready to hand), and so often, it is not considered within health technology projects until it breaks down or gets in the way. Health IT systems are also patch-worked and path-dependent, in which components emerge incrementally and so cannot be replaced wholescale. Star’s [28] conceptualization of infrastructure challenged the common view of technology as stand-alone artifacts, emphasizing the situated and practical work of implementation. Technology cannot be merely designed and installed, but instead, it must emerge from, and build on, the *installed base* (ie, on systems and practices already in place).

In the years before COVID-19, we conducted ethnographic research in 4 NHS organizations in England, all of which were seeking to introduce or scale up video consultations [7,27,29]. The case studies varied in size, geography, patient population, and the use of digital technology. Common across all was the way in which the “boring things” of infrastructure (eg, internal procedures; locally endorsed standards; aspects of software functionality; mundane administrative issues, such as room bookings; general pressures on the system; and interoperability and compatibility issues across new and legacy systems) greatly influenced the fortunes of this initiative.

In our application and retheorization of Star’s [28] notion of infrastructure, we identified several infrastructural features related to the limited uptake and use of video: (1) intricacy and lack of dependability of the installed base; (2) interdependencies of technologies, processes, and routines; (3) the inertia of established routines; (4) the constraining (and, occasionally, enabling) effect of legacy systems; and (5) delays and conflicts relating to clinical quality and safety standards [27].

A crisis is a low-probability, high-impact event that threatens social and life-sustaining systems, creates deep uncertainty, and requires international and government intervention [30]. Gkeredakis et al [31] applied 3 different perspectives to shed light on the varied uses of digital technology, and associated tensions, during the COVID-19 crisis: *opportunity* for accelerated innovation and removal of barriers to experimentation; *disruption* to organizational and occupational practices, generating new dependencies and risks; and *exposure* of vulnerabilities in both people and infrastructures that have previously gone unnoticed. Gkeredakis et al [31] observed that although the pandemic accelerated and expanded the use of digital technology, these shifts were “fast-paced, dramatic and not well understood.”

In this paper, we provide longitudinal qualitative and quantitative data (spanning before and during the pandemic) on 4 NHS organizations to understand infrastructural changes during the rapid scale-up of video consultations and how this may influence sustainability going forward. All 4 sites saw a significant reduction in face-to-face appointments, and immediate and substantial increase in remote ones (overall a 245-fold increase in video consultations), as part of a systemwide response to the pandemic in 2020. Against this background, we sought to study how health information infrastructures shaped, and were shaped by, the rapid scale-up. In the remainder of this paper, we first describe the national context and aims of the study. In the Methods section, we describe the study setup and our analytical approach to understanding infrastructural features and challenges. We then describe our findings on crisis-engendered change as a time of opportunity, disruption, and exposure. Finally, we discuss the findings in the context of the wider literature, highlighting learning points for scaling up and sustaining the use of video beyond the pandemic.

### National Context

Since 2010, there had been a growing policy emphasis on digital innovation and remote care in England [32-34]. Prepandemic, this provided impetus for innovation-driven change. However, the adoption of video consulting was slow, time-consuming, and resource intensive, with activity confined to specific clinical services and settings (typically with a local clinical enthusiast leading) [7,35,36].

In 2019, the year before COVID-19, NHS England and NHS Improvement (NHSEI, the national implementation arm of the NHS in England) set up several pilot video consulting services in secondary care using a video platform called Attend Anywhere (building on the learning of a similar program in Scotland) [37]. Attend Anywhere is an internet browser–based video technology that can be accessed by a staff member on a work computer or a member of the public using their own device. One defining feature is its inbound workflow, which seeks to emulate how patients attend in-person appointments. For example, a single button on a website (or a consistent weblink address on an appointment letter) offers a one-stop virtual front door for patients. On clicking that link, the patient enters a virtual waiting area, before being invited into the clinician’s virtual consulting room.

When the pandemic hit in March 2020, the use of remote consultations (phone and video) formed a key element of the national response [38]. Building on the national pilot of Attend Anywhere, central procurement of the software was extended to provide all NHS trusts unlimited use of the software for 12 months. Staff training and materials to support swift deployment were made available through the NHS England website [39]. National, regional, and interorganizational materials were also shared through the FutureNHS platform (a virtual networking platform for health and social care staff). Within 3 weeks of the pandemic, the NHSEI established 7 regional implementation teams (to provide temporary setup support, webinars, and peer learning) and a national helpdesk (leveraging NHS England’s existing IT call center). Alongside training and operational resources, every NHS trust in England was provided £20,000 (US $ 23120.40) capital funding to purchase video call equipment, and 5000 iPads (Apple Inc) were rapidly sourced and distributed for frontline staff.

NHSX (a cross-department unit within the NHS with responsibility for setting the national policy on digital and data management) advised on governance reviews of video platforms to facilitate organizational approvals, as well as the negotiation of zero-rated 4G with major mobile network providers (so patients could use Attend Anywhere without incurring mobile broadband charges).

The provision of Attend Anywhere across England was further supported by recent insights from prepandemic scale-up of Attend Anywhere in Scotland. In addition, the temporary use of the Scottish platform servers during the initial response allowed for immediate setup of the trust’s Attend Anywhere accounts before they were transferred to a dedicated server.

Across England, the proportion of remote consultations (phone and video) surged from just 3.9% of all outpatient activity in January-February 2020 to 36.6% by the end of April 2020. Over the course of 1 year (April 2020-March 2021), remote consulting accounted for, on average, 28% of outpatient activity (with a proportionate drop largely due to a gradual increase in the number of in person appointments) [40]. Despite this increase in remote consultations, video constituted a relatively small proportion of the appointment activity, in which it made up approximately 2.4% of overall secondary care consultations, although with much variation in uptake across settings and specialties [41]. However, this presented an unprecedented shift in the scale of video consultations, which is considered to have a long-lasting role going forward [42]. For further details on the varied UK context, see Shaw et al [43].

The aim of this study was to draw lessons from 4 NHS organizations on how information infrastructures shaped, and were shaped by, the rapid scale-up of video consultations during COVID-19. Our research question was, “How did information infrastructures shape, and become shaped by, the rapid implementation and scaling-up of video consultations during the pandemic, and what does this mean for scale-up and sustainability going forward?”

## Methods

### Study Design

A naturalistic case study with an action research component was conducted in 4 NHS trusts in England, which we refer to by their pseudonyms: Petroc, Eastern, Southern, and Northern Trusts. All were seeking to introduce and scale up video consultations before and during the pandemic as part of a service improvement program, in which members of the Petroc team supported clinicians and managers in the 3 other trusts. Data sources included ethnographic field notes, interviews, consultation activity data, service evaluation reports, documents, and material artifacts.

### Ethical Considerations

Research ethics approval was obtained from London – Camberwell St. Giles Research Ethics Committee (ref. 19/LO/0550). An advisory group was established from the start to oversee both phases of the project, with wide stakeholder representation (eg, policy makers, organizational stakeholders, and patient groups) and a lay chair. The group provided input on study progress during 6-monthly meetings, as well as comments on project outputs by phone and email.

### Data Collection and Management

Data were collected in 2 phases, before and during the pandemic (the periods and data sources are presented in Table 1). The prepandemic phase extended data previously reported up until when COVID-19 was confirmed in the United Kingdom (January 2018-February 2020), providing context to the impact of the pandemic. The in-pandemic data were collected during March 2020-July 2021.

In total, 90 interviews were conducted with 49 participants, including doctors, nurses, allied health professionals (AHPs), service managers, and admin and IT support. This included 43 (48%) interviews with 29 (59%) participants prepandemic and 47 (52%) interviews with 37 (41%) participants during the pandemic. In addition, 17 (35%) participants were interviewed in both phases, and 6 (12%) key informants were interviewed on multiple occasions within each phase.

Fieldwork was conducted in person before the pandemic but was, of necessity, conducted remotely during the pandemic. Interviews lasted between 30 and 60 minutes. Participants were asked to talk about their experience of supporting or using video consultations (or why they had chosen not to support/use this medium), including the progress of and the challenge in using (or supporting the use of) video and the impact of the pandemic. When interviewees talked in the abstract about problems and challenges, we asked them to describe specific examples of these.

Our data set of qualitative interviews was supplemented by evaluation data captured by local teams (eg, aggregated data on patient experience surveys and demographics), internal audits (staff engagement reports), policy documents (eg, digital strategy documents, information governance, service recovery plans), and standard operating procedures and training resources (eg, implementation procedures, guidance materials).

Video consultation activity was captured during March 2020-March 2021 as part of a national NHS England pilot using Attend Anywhere, which was the main video platform used within the sites.

**Table 1:** Data sources for the 2 phases of the evaluation.

		Phase 1 (prepandemic)	Phase 2 (during the pandemic)	Total
Period of data collection		January 2018-February 2020	March 2020-July 2021	3 years, 7 months
Ethnographic observation (hours)		180 hours	No ethnography possible due to the pandemic	180 hours
**Interviews by site**				
	Petroc	n=22 (54%)	n=19 (46%)	41
	Eastern	n=5 (38%)	n=8 (62%)	13
	Southern	n=12 (52%)	n=11 (48%)	23
	Northern	n=4 (31%)	n=9 (69%)	13
Interview participants		7 doctors 5 nurses 3 AHPs^a^ 8 managers 6 admin/IT staff	10 doctors 3 nurses 7 AHPs 8 managers 9 admin/IT staff	12 doctors 6 nurses 8 AHPs 10 managers 13 admin/IT staff
Uptake statistics for video consultations, by NHS^b^ trust		July 2019-February 2020	March 2020-March 2021	21 months
Online patient survey reports		Postconsultations, user experience survey (Petroc Health)	Postconsultations, user experience survey (all 4 sites)	5 patient surveys (N=4050)

^a^AHP: allied health professional.

^b^NHS: National Health Service.

### Analytical Approach

Interviews, field notes, and supporting materials collected during the pandemic phase were first used to gain familiarity with each case site and produce an organizational narrative on the impact of COVID-19. Following this familiarization phase, interview transcripts were then organized into a Microsoft Excel spreadsheet to identify emerging themes within Gkeredakis et al's [31] 3 perspectives on the use of digital technology in a crisis: as an *opportunity* (for the implementation, uptake, and use of video), as a *disruption* (to the existing working practice and the need to adapt), and as *exposure* (issues that had previously gone unnoticed or underplayed).

Analysis of emerging subthemes was guided by Star’s [44] ethnographic approach to the study of information infrastructure to surface master narratives (the overarching discourses that shape decisions), infrastructural inversion (eg, foreground things that are usually kept in the background), surface invisible work (eg, work done by low-grade staff, such as secretaries and administrators), and study paradoxes (eg, why a simple change makes the whole system unworkable, perhaps because it generates additional hidden work) [44].

Analysis was further guided by the literature on crisis management [45] and routine dynamics [46]. Christianson et al [45] highlighted how the novel demands of *rare interruptions* trigger organizational learning and reveal underlying weaknesses and potential strengths as events unfold. This includes organizational actors engaging in the process of *interpreting* (which initiates the conditions that guide responsive action), *relating* (when members of the organization understand their contribution to the overall outcome), and *restructuring* (reconfiguring social and materials structures to maintain core organizational functions). In relation to the latter, we also drew on the notion of organizational routines, defined as “recognisable, repetitive patterns of interdependent action carried out by multiple actors” [46]. Organizational routines coordinate work, reduce uncertainty, and are situated within a sociomaterial context, structured around time, physical spaces, and material and technological artifacts [47].

Pentland and Feldman [46] distinguish between *ostensive* routines (abstract understanding about how it is enacted) and *performative* routines (the range of ways in which it is carried out in practice). They propose the use of *narrative networks* to represent the generative tension between these 2 aspects of organizational routines and how this is mediated by *technology in use* [48]. Unlike other graphical representations of work, such as process mapping, narrative networks summarize the relations between actions as a networked graph of nodes (categories of actions) and linked edges (indicating sequential relations between the actions), providing a network of potential performances or “stories” within a process. We applied this approach to help us understand how video was used and embedded within routine practice.

## Results

### Case Site Overview of Rapid Scale-Up

Petroc Health is a multisite acute hospital trust located in a predominantly deprived and multiethnic part of London. It is 1 of the largest trusts in England, serving a population of 2.5 million. Since 2013, Skype had been used within a diabetes clinic to reduce did-not-attend rates. Building on the success of this pilot, the digital strategy team commenced a trustwide program in 2018 to spread use across outpatient services. However, progress was hampered by technical problems with Skype, including incompatibility with the new virtual desktop infrastructure (VDI, a virtualization technology that hosts a desktop operating system on a centralized server). Petroc joined the NHSEI Attend Anywhere pilot in October 2019, and 7 clinical services started using the platform by the time COVID-19 hit in March 2020. As part of the pandemic response, other members of the digital strategy team were drafted in to support scale-up (eg, training, deployment), with oversight from a cross-departmental covid executive group.

Southern Trust is a large multisite provider in a university city that had won awards as a digital innovator. It includes 4 hospitals serving a population of 655,000, with a relatively high proportion of young people aged 20-24 years (including university students). As part of the prepandemic scale-up program, 2 services (diabetes and orthopedics) started using Skype for Business. Although this software was supported by the trust network, licensing permissions and firewall restrictions created problems conducting video calls with people outside of the organization (eg, patients). A small group of clinicians sought to engage the information and communication technology (ICT) department into resolving these issues, but little progress was made due to other IT priorities. In July 2019, Southern Trust joined the NHSEI Attend Anywhere pilot, in which 4 services started using the platform. When the pandemic hit, the chief digital officer coordinated a trustwide deployment, with the support of 5 divisional digital leads to manage technical setup (eg, the provision of iPads to run video via secure Wi-Fi).

Eastern Trust runs 2 hospitals in a largely rural county. The catchment population of 1 million is predominantly White British, with a relatively high proportion of patients over 65 years. There was a prepandemic strategy to digitize patient records. However, the electronic patient record system had not yet been rolled out, and so the large majority of services still relied on paper-based records. A diabetes consultant’s initial attempts to pilot the use of Skype, as part of the prepandemic scale-up, were halted by the ICT department due to governance concerns. The trust joined the NHSEI Attend Anywhere pilot in November 2019, but none of the services started using the software until COVID-19. This meant extensive work was needed to prepare clinicians and administrative teams using the software, with training from the regional support team set up by the NHSEI.

Northern Trust provides hospital and community services across a built-up metropolitan borough, as well as small towns and rural areas. Although geographically the largest catchment area, it has the smallest patient population of approximately 500,000. The population is predominantly White British, with socioeconomically diverse regions, including areas of high deprivation, unemployment, chronic illness, substance misuse, and mental health problems. As an early participating site for the NHSEI Attend Anywhere pilot, prepandemic use of the video software began in July 2019, with a focus on reducing patient travel and strong senior management buy-in. By the time the pandemic hit, Attend Anywhere was being used in 6 clinical services. The implementation team continued to lead the deployment, with additional operational and training support brought in from the IT and business units.

**Figure 1:**
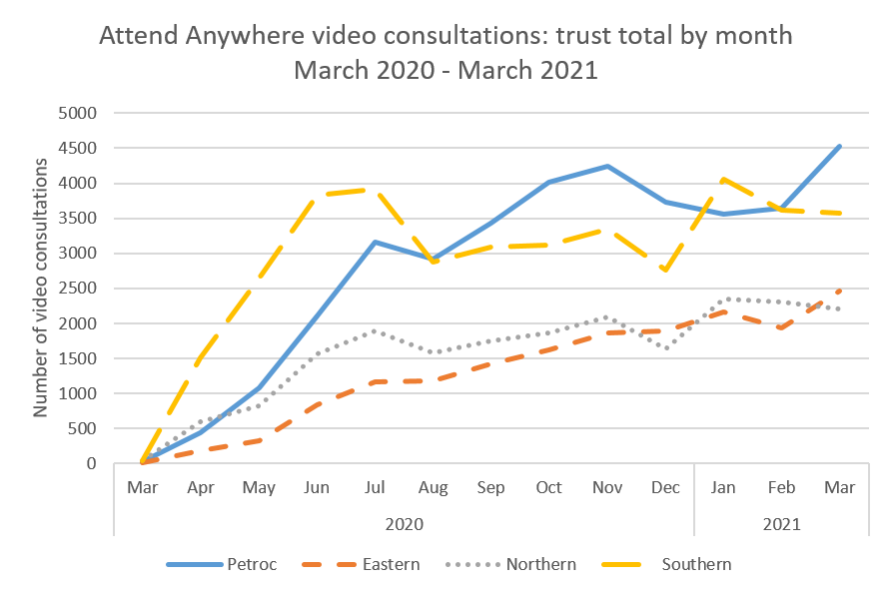
Monthly video consultations across the 4 trusts during March 2020-March 2021.

Figure 1 shows the number of video consultations for the 4 sites during the period of March 2020-March 2021. The most rapid increase in the use of video was seen during the first month of the pandemic (March-April 2020), from an average of 8 video consultations per week to 171 per week. Uptake continued to increase over the course of the pandemic, averaging approximately 800 video consultations per week by March 2021. As was observed nationally, video still constituted a relatively low proportion of overall outpatient activity, in which most remote appointments were conducted by phone.

In sum, the case studies present contextually different circumstances in relation to the geography and technical and organizational contingencies but show similar trends in the rapid scale-up of video consultations. The following sections highlight common themes in relation to how the information infrastructures shaped, and became shaped by, the rapid scale-up.

### Opportunity

Viewing the pandemic as an opportunity for positive change highlights how the crisis helped accelerate processes that were stalling, questioned institutional norms and processes, and allowed for experimentation.

Before COVID-19, efforts to implement video were restricted by (to a varying degree across sites) pressures on human and financial resources, competing operational and strategic priorities, differing institutional logics (beliefs, assumptions, and practices that shape actions [49]), problems interfacing the new technology with local legacy systems and standards, and training and support requirements for staff and patients. As the project manager within Petroc expressed shortly before the pandemic:

It’s difficult to make a financial case for video consultations, because we’re not reducing any activity in the system, but we are requesting new technology…And it is difficult timing for the trust to take on something that doesn’t even pay for itself…You need the senior leadership to see it as the next priority, but of course, there are hundreds of priorities. It is really challenging. It doesn’t happen overnight…

Bridget, program manager, Petroc Health, January 2020

The prepandemic focus on the economic case echoed the dominant policy discourse that viewed digital solutions primarily in terms of enhancing efficiency [33]. Other confounding logics included frontline duty of care (professional standards of what excellent care looks like and any potential risks of harms the digital medium might bring), IT regulatory concerns on data security and service quality, and managerial focus on capacity and resource to support the change. In this context, the translational efforts were typically driven by local clinical champions’ ability to define and frame the problem for which video was to be a solution, engage interest, and mobilize other key organizational actors to support the initiative [27]. Broadly speaking, such efforts were rarely successful and even more rarely sustained long term. Video consultations could be established as small-scale demonstration projects but almost never gained momentum as business as usual.

However, in the wake of the pandemic, a national mandate to avoid direct clinician-patient contact on the grounds of safety (infection control) engendered collective action toward the rapid rollout, as the same project manager described:

All of a sudden, we have a level of support we never had before. Like from a very senior level. As soon as this [pandemic] happened, they put in temporary governance structures—the Pandemic Covid Outpatient Group—headed up by one of the most senior doctors in the trust. I’ve moved full-time on this and report to them. So, if I have any problems, I have someone to escalate to, who can do something about it…Also, the clinical system team [responsible for building electronic clinic schedules] usually take[s] weeks to implement changes. But I’ve been given permission to, basically, get in touch with them to say, “This is a priority.”

Bridget, program manager, Petroc Health, April 2020

These new relations illustrate how a shared *organizing vision* (clear and consistent vision among stakeholders as to what will be achieved) [50] brought legitimacy and support for the change, in which staff and resources were mobilized to address infrastructural constraints. As illustrated in the extract earlier, provisional management and communicative structures helped hold groups together around this narrative and increase awareness of their role within the context of the collective outcome.

An opportunity was also seen within the temporary suspension of regulative structures, allowing staff to bypass administrative burden and governance processes, which had previously thwarted attempts to introduce video:

Things kind of became, to an extent, easier to do. Previously, you would have a lot of bureaucratic hurdles and hoops to jump through. But we have come to a point where things just had to be done.

Nathan, operational support, Northern Trust, March 2021

This brought a welcome cultural shift, providing greater autonomy and openness to change. In accordance with Scott’s [51] 3 interacting institutional forces that operate in health care, an easing of risk management protocols (*regulative*) enabled more agile, unformalized (*cultural-cognitive*) governance, underpinned by professional (*normative*) values:

Before [COVID-19], we had hearing aids where you could remotely program them, and I looked into setting up a remote programming clinic…I went to information governance, and they gave me this list, and I was like, “Oh it’s not worth it—not for a few patients.” So, I didn’t bother. And that’s terrible—I should have bothered. But it was too much work. But now, when we asked them, it went straight through to the medical director, and she just said, “Go ahead with it…” So, things progressed—which is what the NHS would struggle with sometimes.

Karen, consultant, Southern Trust, November 2020

As described before, regulatory constraints were also reduced through national measures by NHS England, including the central procurement of Attend Anywhere (enabling unrestricted use with no cost to trusts), endorsement of video platforms by national information governance bodies (enabling experimentation), and temporary tariffs for remote consultations (avoiding local commissioning requirements and associated system configurations).

However, although these were intended as *temporary* arrangements to deal with the pandemic, they set organizations on a particular infrastructural path, upon which they were now destined to build:

One challenge is, few of us want to change what we’ve got now [Attend Anywhere]. We will have to go through this procurement—because the region will be doing it on our behalf—and there is a slim chance another provider will be chosen, and that would be a real challenge for us.

Teresa, program manager, Eastern Trust, February 2021

Finally, the pandemic created an opportunity for clinician engagement. Before the pandemic, there was a striking difference between those who embraced the use of video with enthusiasm and other clinicians on the same teams who were reluctant or could not see the benefits of the change. Hurdles to adoption were less to do with needing to learn to use the technology and more related to professional concerns about patient safety, quality of care, and identity. However, during the pandemic, the perceived *relative advantage* [52] of video compared to existing alternatives meant that it was no longer seen as a suboptimal option:

There was quite a lot of resistance previously by medical colleagues. They felt it would affect the relationship. [COVID-19] has turned that on its head quite quickly.

Eleanor, diabetes nurse, Southern Trust, June 2020

Clinicians using video talked about the advantages over phone for basic visual assessments (“eyeballing” the patient) and nonverbal interaction. This was reflected in the video activity data across sites, in which a large proportion of consultations were conducted within psychiatry/psychology and mental health services (13,952/109,401, 12.75%, of overall video activity), pediatric services (12,964/109,401, 11.85%), musculoskeletal and orthopedic services (11,953/109,401, 10.93%), and physiotherapy (11,636/109,401, 10.64%).

Crucially, many clinicians described *reflection in action* [53] through the pandemic, in which the use of video reshaped prior assumptions on the role of such technology within their clinical practice:

You have to be very pragmatic [over video] about how you do your assessments. And all those special tests we used to do in the consultation room, we’re suddenly finding that we were not getting any value out of them. And we are now coming to realize, it is what the patients tell us, it is how they got out their chair to get their medication or how they turn around to tell their partner to turn the telly down—all these little functional cues that we are seeing. I’m finding video a lot more helpful than these clever tests we used to do.

Larry, physiotherapist, Northern Trust, August 2020

Christianson et al [45] describe how the uncertainties generated by rare events provide such opportunities to question previously held assumptions about core organizational functions and roles and provide a *strategic opening* for supporters of the initiative. Other opportunities and extended uses of video included remote multidisciplinary and group consultations, which would have previously been difficult to establish as physical encounters.

However, although the pandemic provided a context to try out and reflect on the use of video, as predicted by Gkeredakis et al [31], an opportunity through a crisis also comes with inherent tensions going forward. A key challenge raised related to subsequent shifts in the social and political forces as the risk of COVID-19 decreased:

The big question for me is whether this change is permanent or just temporary…Are we going to see clinicians just not wanting to go back to the way they were working before, or are we going to see a slip in the use?

Beth, program manager, Northern Trust, April 2021

The absence of prepandemic groundwork within Eastern Trust also brought uncertainties about the extent to which the new service model can be socially and technically stabilized within the organization:

We built a bridge—and it was a pretty good bridge to jump over the river—but we didn’t build the foundations. We suddenly had the technology, instantly threw ourselves into it, and got over that river. But the bridge is a little insecure…As a major change project, you would never do that—implement a new technology, try to get hundreds on board, and then build the system to support it.

Teresa, program manager, Eastern Trust, December 2020

### Disruption

Viewing the pandemic as disruption highlights how staff had to adapt to widespread displacement of social and material environments, including the need to work from home. Technology shortages also demanded improvised use of computing equipment (an advantage of Attend Anywhere was that it could run on a personal device, as nothing needed to be downloaded). Staff needed to reorganize work routines to accommodate and support remote consultations in the face of prolonged uncertainty and disruption. Adaptive capabilities allowed some service provision to continue but also distorted work practices and created new demands and unintended consequences.

Clinic workflows are complex and structured around various interacting routines (eg, booking appointments, arranging prior tests, processing patients through the clinic) and associated spatial and material structuring devices [54], which have largely evolved around the *physical* copresence of patients and staff. Indeed, the inertia of established routines was a major constraint on the use of video before the pandemic, in which apparent embedding resulted from elaborate workarounds. Hence, the sudden and extensive transition to *virtual* created significant disruption, in which a fix for one problem generated problems elsewhere in the system.

Particularly during the early stages of the pandemic, clinicians relied on the telephone to consult with patients:

We turned all into blanket telephone—and in a sense that has been the legacy—and we haven’t moved forward with video clinics, because the telephone clinics have been kind of working…There is a logistical problem. If you see people at clinic, it is really easy because they turn up, there is a slip [of paper] with their name on the trolley, and you just come out, pick up the slip…And when you’ve seen them, they will leave—you can’t double-see them. With the phone, we don’t have that. You may get someone who does not answer, so you call the next person, and come back to them—but then of course, that next person wasn’t expecting a call at that time, so then doesn’t pick up…So at the moment, I’ll start the [in clinic] appointments, and the registrar starts on the phone list. And then, when there are some less complicated patients in clinic, the registrar sees them, and I start at the bottom of the phone list and work up.

Frank, neuro-oncology surgeon, Petroc Health, October 2020

This extract highlights how the “installed base” both enabled and constrained the rapid reorganization of clinic routines, which, in turn, became locally embedded. Feldman [55] describes how such “patterning” (the process of reinforcing old and creating new patterns simply by taking action) initially emerges through the stringing together of anticipated-orientated actions (situated and highly influenced by the fit between available opportunities and their current abilities) under prolonged disruptive conditions. Although these “provisional adjustments” are enacted to make a situation work, over time, they form new patterns, roles, and ways of doing things.

Similarly, the rapid expansion of video demanded a high degree of adaptation and emergence in accordance with local needs and contingencies. One of the main areas of focus was the management of patient “entry” into their video appointment. The main video platform (Attend Anywhere) uses a consistent URL for each clinic virtual waiting area. In the initial phase, most patients were sent the relevant waiting area URL on an appointment letter, which they would be required to write into their internet browser. Although quick to implement, this arrangement was prone to problems with typing errors and incomplete knowledge of the new process:

They send them a sheet. I guess it must say what they have to do, but it’s very limited. In fact, I don’t think they always even send that. Sometimes, they just send a letter saying log onto this [URL] address. And 9 times out of 10, the first time they use it, they’ll go onto Google Search…which just comes up with [Attend Anywhere] websites. And then, we say ‘No, you put it into the address bar at the top…”

Betty, audiology therapist, Eastern Trust, February 2021

In this extract, the clinician, patient, and administrative staff lack the mutual awareness [56] and embodied knowledge needed to coordinate actions within this new digital space—aspects that are deeply embedded and taken for granted in traditional outpatient waiting areas.

However, over the course of the pandemic, various changes were made to help orientate patients, depending on local human and technical resources in place. Within Petroc, for example, existing (outsourced) text reminding services adapted the text message sent to patients so they could click directly onto the URL, although this relied on the patient having a smart phone.

Another route of entry, eventually established within Petroc, Northern, and Southern Trusts, was the trust website, in which patients could click a button for the relevant virtual waiting area, although this relied on the patient being able to navigate the website and locate the correct button. The following extract describes a common problem in which patients would turn up in the wrong virtual waiting area due to difficulties distinguishing between overlapping specialties. This created a high degree of “invisible routine articulation work” (work necessary for dealing with anticipated contingencies but that is not formalized or documented [57]) for the clinician to locate and orientate them accordingly:

Patients select a waiting room to go into. But often, they would be waiting in orthopedics because they had seen that department previously…But we don’t have access to that waiting room. So, we would need to contact them and tell them to come out and go into the MSK [musculoskeletal] waiting area.

Nick, physiotherapist, Petroc Health, March 2021

Organizational capability to endure, and even harness, locally driven adaptations depended on the people and connections in place to monitor and embed new practices. For example, the Northern Trust implementation team continued to engage with clinicians shortly after the rapid deployment of Attend Anywhere, during which they discovered some practitioners using an alternative platform (accuRx). This platform’s texting and video call functions aligned better with their particular workflows and systems, and so it became supported at the system level.

It was kind of by chance and through discussions that we found some people had started using [accuRx]...We realized it was building a bit of momentum amongst some clinicians. So, we officially approved it, did a bit of comms, and developed some training on how to use that platform.

Nathan, operational support , Northern Trust, March 2021

Similarly, over the course of the pandemic, the developers of Attend Anywhere incorporated new design features based on user feedback, including the “consult now” function, allowing clinicians to send the URL link directly to patients via text or email.

The various sociotechnical arrangements to support patient entry reflect the generative tension between ostensive (generalized understanding) and performative (specific actions taken) aspects of routines [46], driven by clinicians’ efforts to balance efficiency (managing their time and capacity) with flexibility (to enhance patient access) in this new virtual space. Pentland et al [58] highlight the paradoxical tension of *process multiplicity* (a single process incorporates many possible “paths”), in which ICT greatly expands the “space of possible paths”; different ICTs may be used at various points within the same process. Even a simple change (eg, adding a new button to enter a virtual waiting area) creates a host of new actions and pathways.

**Figure 2:**
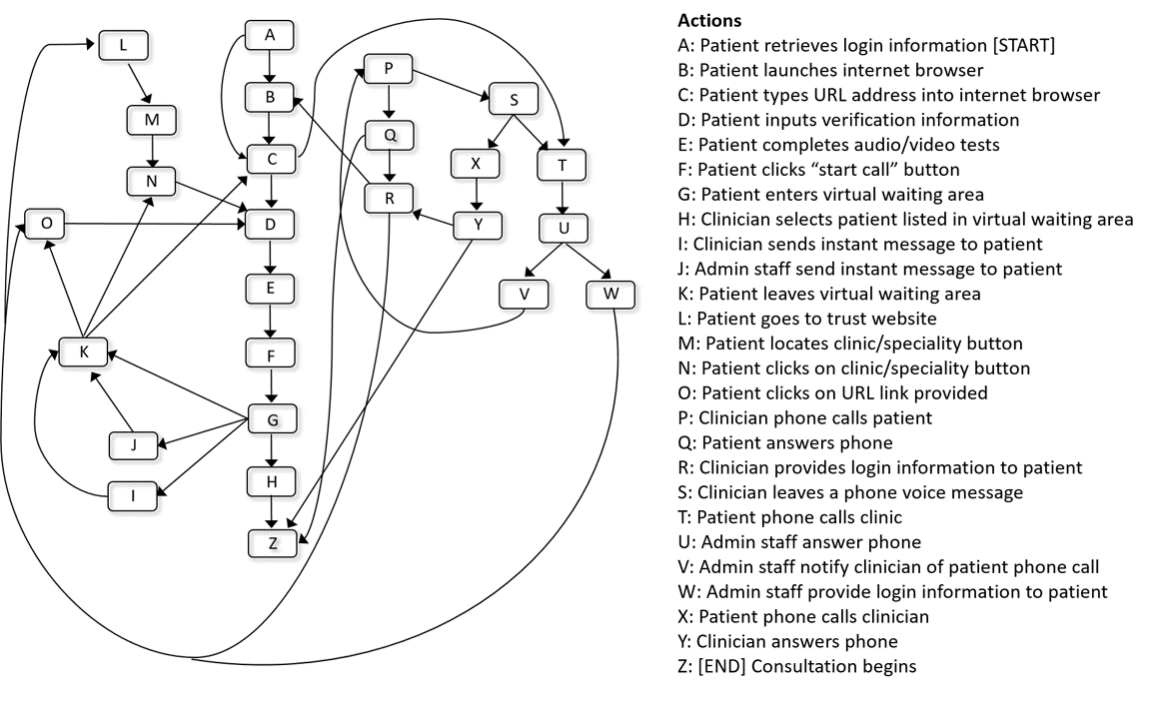
Narrative network analysis of patient entry into video consultations.

Figure 2 presents a narrative network of known possible actions involved in the process of patient entry into a video appointment, based on participants’ accounts of using Attend Anyway. Seeing the process as a network of related actions highlights the extent of performative variation within this component of the workflow. Each step leads to various alternative actions that could happen next, enabled by the video software and associated technology. For example, some clinicians talked about a tendency to phone the patient if they were not in the virtual waiting area. From there, they might explain to the patient how to access it, or send login details electronically. Sometimes, patients would not answer the phone, so the clinician might leave a voice message (if the technology allows); some would also leave their direct contact number (as caller IDs were often withheld). Upon hearing the voice message, several actions could occur next; the patient may attempt to (re)connect to the virtual waiting area, they could phone the clinician back, they might call the clinic reception, or they might do nothing.

Another example of process extension was clinicians’ use of the instant messaging feature to communicate with patients in the virtual waiting area (eg, to tell them whether the clinic is running late), in which patients may decide to wait or exit and re-enter at a later point. In some cases, the clinician and patient would jointly decide to abandon the video option and consult by phone instead (when clinically appropriate).

Although some service teams brought admin support into this process (eg, to monitor the virtual waiting area), it usually relied on the clinicians to manage. Participants talked about the need for a conceptual shift in the departmental management of these new digital spaces, if they are to be sustained:

If the patient has a face-to-face appointment, it isn’t just them instantly in with a clinician and instantly out with a clinician…Outpatient departments manage the physical outpatient space, but now we’ve got all of this virtual outpatient space…which actually…other than the patient and the clinician linking up together, nobody is managing that space to make sure that link-up happens correctly…We need to be taking responsibility, full responsibility—like we do for the physical space—of that virtual space.

Simon, ICT manager, Northern Trust, March 2021

### Exposure

Perspectives on exposure revealed infrastructural challenges and tensions to video consulting that have gone largely unnoticed. As Star [44] points out, infrastructure tends to exist in the background, invisible and taken for granted until it breaks down or gets in the way. Before the pandemic, clinicians using video described their use as being ad hoc, on a small scale, and with selected patients, rather than business as usual. The unprecedented demands of the rapid scale-up effort exposed important social, technical, and ethical issues to mainstreamed use—what Christianson et al [45] refer to as the “brutal audit of weakness” brought about by the novel demand of rare events.

Networks and servers came under significant strain during the first few months of the pandemic, resulting in periods of poor service reliability. Although these problems were dealt with promptly in collaboration with technology providers, local IT issues were poorly understood and more difficult to resolve:

It is difficult to test the consultation capacity, because it is very dependent on the volume of calls, how the platform is doing on that day…It is really difficult to test the actual performance because it can vary so much.

Tanvi, operational support, Petroc Health, January 2021

Additionally, access and dependability issues extended beyond the boundaries of the organization’s IT infrastructure, including “digital poverty” (eg, no smartphone, no webcam, limited data package). It was reported that video tended to be lower among groups with a greater health care need and already facing health inequalities, such as older people, low health literacy, weak social networks, limited mobility, limited transport, and psychological and mental health problems:

Some people don’t have [smartphones], they don’t have laptops, iPads. You have to be kind of fairly well off to be able to access video consultation. We’ve definitely seen a difference in what type of patients can and can’t access.

Nick, physiotherapist, Petroc Health, March 2021

Clinicians talked about limited privacy in family households, as well as differences in network connectivity. Particularly for patients living in rural areas, audio-video quality was unreliable, impacting the quality of the consultation and sometimes resulting in the use of the telephone as a backup:

Because if you’re talking to someone and you’re trying to explain something or you ask them a question and then you have to wait 10 seconds for them to answer, then you think, “Are they not answering, or is it they’ve not heard me or, you know, is it the delay?”

Betty, audiology therapist, Eastern Trust, February 2021

It was often difficult to anticipate the needs and wishes of patients in advance, requiring flexible use, alongside nondigital alternatives. Digital exclusion was not merely down to whether a person could use technology, but cut across multiple technical and social elements, as described next:

I had to say to her, “I can see your background [on video].” And I don’t think she was comfortable with that…We have a large Asian population, where there is an element of larger families and smaller living spaces. If I access their space, I will not only be seeing them, I would be seeing their family. And they may not be comfortable with that…You have to be careful.

Tanisha, hepatologist, Petroc Health, October 2020

Mitigating digital exclusion was an effortful accomplishment between clinicians, patients, and their support networks. However, some early strategic work had begun within Northern Trust to establish local hubs for video appointments in partnership with community centers:

So now, if they need help accessing technology, they can get in touch with the community center—and we trained people in these community hubs to help people access the remote consultation. But also, in the community hub, they will get access to a full package of support, such as food packages…It has taken a while just engaging with these groups, making sure they are happy.

Tessa, PPI engagement lead, Northern Trust, April 2021

This extract reveals the role of collegial partnerships in order to coordinate and interface between the digital and material aspects of health care and community-based infrastructures. Perspectives on exposure, therefore, reveal not only weakness within the system but also strengths to be leveraged and built upon. In a similar vein, Sanner et al [59] draw on the horticultural notion of grafting to describe how infrastructural mergence between existing systems (from separate organizational boundaries) can be supported through the mutual adjustment and careful alignment of available resources and interests. Such perspective draws attention to how health IT organizations may need to interface with other organizational and professional boundaries and associated infrastructure in order to enhance the accessibility of remote digital care.

## Discussion

### Principal Findings

Gkeredakis et al's [31] 3 perspectives on crisis and digital change provide different vantage points to foreground how health IT infrastructures shaped, and were shaped by, the rapid scale-up of video consultations. Considering COVID-19 as an *opportunity* highlighted the institutional elements of infrastructure, in which conventional assumptions, norms, and governance structures were challenged. The pandemic brought about a collective understanding or *organizing vision* [50] for video, in which the organizational application shifted from a focus on efficiency and access to an issue of safety (infection control). This played a key role in legitimizing the technology and mobilizing resources to support adoption at scale. Beyond the pandemic, the organizing vision remains uncertain. Although many perceive a long-term role for video, the balance between the benefits and harms of such a service model will change, as the context moves from one dominated by risk of infection to concerns about the impact of remote consulting on patient safety and quality of care.

The focus on *disruption* drew attention to the structures and practices involved in the urgent response to the pandemic. With the displacement of deeply embedded routines, new performative patterns of action emerged, in which the virtual environment greatly expanded the “space of possible paths” within consultation workflows. A large space of possible paths makes routines more resilient to disruption (by minimizing the single points of failure), but it also makes the processes more difficult to replicate and manage [58]. The capacity to retain and mainstream positive elements of the change beyond the pandemic will require a judicious balance between the managerial top-down (clear processes and milestones) and the emergent bottom-up (responsive and contingent) approaches.

Accounts related to *exposure* reflected inherent properties of information infrastructures, as backgrounded and taken for granted. The accelerated scale-up of video revealed social and technical impediments to video consulting and the potential to accentuate unresolved health inequalities. This raises practical and ethical challenges to scaling up such a service model and conflicts with the policy talk of such technology as state of the art, efficient, and accessible [33]. Improving access will be an extensive and ongoing accomplishment, requiring close attention to the materiality and dependability of the installed base, as well as the social and cultural context of use.

### Enabling Infrastructure Growth in the New Normal

Research on information infrastructures highlights how technology-supported change needs to be cultivated in a way that acknowledges challenging organizational needs and the inertia of the installed base [60-62]. The extreme and stochastic nature of the pandemic has provided a unique insight into the processes and mechanisms that surround infrastructural change, including the path-dependent way in which the system is built and added to, and, in turn, set organizations on a particular infrastructural path for remote consulting.

Despite the policy focus on digital [63], the reality is that most remote consultations were conducted using the telephone. The pandemic nevertheless brought about a significant shift in the use of video, which shows promise for particular clinical contexts. Building on our previous research on the challenges in introducing video consultations in health care settings, this study provides further insights for development and sustainability going forward.

First is the need to balance stability (and integration, security, and centralized control) on the one hand and openness to change (and emergence and local adaptability) on the other. Although regulatory changes during the pandemic may have brought a new-found agility to experiment and innovate, health care organizations must still ensure a high level of safety, security, and dependability [8]. Bygstad and Iden’s [64] notion of *bimodal* governance offers a potential approach, in which “heavyweight” IT systems (traditional and sequential, emphasizing safety and accuracy) and “lightweight” IT applications (exploratory and nonlinear, emphasizing agility and speed) are managed separately under differing organizational structures, cultures, and regulatory mechanisms. Such a model has been shown to work well in hospital settings, in which a partitioning of IT departments—one part dealing with the traditional core IT system and the other with new applications—allowed for a “two-speed” approach to the co-evolution of these loosely couple subsystems [65].

Second, the extended use of remote consultations highlights a need to conceptualize information infrastructures beyond the boundaries of the health care organization. The ubiquitous use of extra-organizational ICT within professional work settings has prompted calls for more theoretical and empirical research into the intersection between institutional infrastructures and individual digital assemblages [66]. Video consulting during the pandemic has been found to occur across multiple settings outside of the usual organizational rules and sociomaterial arrangements [67]. Accordingly, our study suggests that more accessible, inclusive, and dependable approaches to remote care require a focus on how health IT infrastructures can merge with other systems across professional, technological, and organizational boundaries. This occurred at multiple levels within our study, including individual configurations (eg, improvised use of personal computing devices), interorganizational partnerships (eg, establishing community hub video facilities), and national strategies (eg, temporary use of Scottish platform servers, negotiations with mobile network providers). Such infrastructural “grafting” [59] involves the ability to identify opportune moments and parts of the installed base to leverage, and work effectively with stakeholders who retained some control over those parts of the infrastructure. This aligns with other studies that have focused on equity and virtual care during COVID-19, drawing attention to the need for a multilevel approach to improving access, including policy level strategies (eg, investment in internet and device access), organizational system design (eg, training and capacity building programs to support users), and community engagement (eg, digital literacy education and supporting resources) [68,69].

Third, close attention needs to be paid to routines and patterns of actions underpinning virtual appointments. Our findings on the disruptive forces of the pandemic resonate with other studies focusing on the redesign of care pathways using process-mapping methods [70,71]. Traditional workflow-style process maps can be useful in many respects. However, our analysis highlights that *virtual* consultations vastly expand the set of possible paths within an appointment process, and therefore, it is important to visualize and understand the multiple performances that *could* be generated in a process (as opposed to a single version of how the process *should* occur). Narrative networks may provide a useful methodological device to see the terrain of possible paths and purposefully increase variation in performance (for flexibility) or try to reduce it (for standardization and control). This could help identify areas to increase resilience and choice (eg, choosing between different remote consulting modalities or platforms), while also ensuring associated roles and processes are consistent and coordinated.

Finally, it is important to bridge competing institutional logics with an overall narrative or organizing vision [50] for video, through ongoing cross-stakeholder dialogue, involving policy, organizational, and patient engagement. The pandemic saw the emergence of intra- and interorganizational “communities of practice” [72] to share experiences and validate knowledge on the use of video. However, by their very nature, communities of practice tend to be informal and unstructured and so difficult to establish and sustain. As we have observed elsewhere [37], quality improvement collaboratives (structured approaches to meeting, sharing resources, and evaluating and informing strategic change) can help promote such learning, leverage national resources, and inform institutional elements of the infrastructure (eg, policies, regulation, funding, training). The varied use of video across clinical specialties highlights a need for further work in this regard to support shared learning as to how, and in what circumstances, this modality works well for patients. In addition, from a managerial standpoint, it will be important to help various organizational actors envisage how their actions interrelate and contribute to the overall narrative or collective outcome. Osmundsen and Bygstad [73] highlight the importance of sense making (process by which organizational actors engage in retrospective and prospective thinking to interpret reality [74]) and sense giving (process whereby organizational actors attempt to influence the meaning construction of other organizational actors [75]) to support a collective understanding of ongoing infrastructural changes. To guide the growth and expansion of remote consultations, it will be important to understand and foster communicative dynamics that promote “collective minding” [76] across various aspects of the supporting infrastructure.

### Strengths and Limitations

The strength of this study is that we undertook research at 4 sites before the pandemic, through which we had already identified the importance of information infrastructures on the implementation and use of video. This provided a unique opportunity to study crisis-engendered changes from the early stages. In addition, the case studies were conducted within a wider program of research on remote consultations across the United Kingdom, providing a wider national context to the organizational settings.

Pandemic restrictions meant that we could not undertake ethnographic work during this phase, and our data collection during this time was affected by the unprecedented pressures on NHS staff, raising potential sample biases toward those individuals with time available to speak with us. However, our positive and longstanding relationship with these sites helped mitigate such issues; the researcher had previously visited all sites on numerous occasions before the pandemic, adapted interview schedules, and was able to draw on multiple sources of data.

A further limitation of this study includes the question of how far we can generalize from our 4 case studies on the rapid scale-up of video consultations. Our theoretical analysis helped explain the empirical data on information infrastructures in our case sites but may not explain all aspects of such infrastructure in all contexts. We chose to focus on video consultations as a topic of academic interest because it exemplifies a promising innovation that has taken decades to scale up. We encourage others to apply the same theoretical lens to explore crisis engendered changes in other digital health contexts.

### Conclusion

Gkeredakis et al’s [31] 3 perspectives on crisis and digital change (as opportunity, disruption, and exposure) provided a useful lens to understanding the role of information infrastructures during the rapid scale-up for video consultations, as well as the challenges and tensions going forward. Foregrounding crisis-engendered change helps explain how the infrastructures constrained and enabled the rapid scale-up and highlights how health care organizations can build on the gains going forward. To extend and sustain the use of video in the long term, it will be important to enable incremental systemic change through agile governance and knowledge transfer pathways, allow greater flexibility and process multiplicity within virtual clinic workflows, attend to the materiality and dependability of the installed base both within and beyond organizational boundaries, and maintain an overall narrative within which the continued use of video can be framed.

## Abbreviations

AHP: allied health professional
ICT: information and communication technology
NHS: National Health Service
NHSEI: NHS England and NHS Improvement
